# One-step synthesis of hierarchical [B]-ZSM-5 using cetyltrimethylammonium bromide as mesoporogen

**DOI:** 10.3906/kim-2001-42

**Published:** 2020-06-01

**Authors:** Büşra KARAKAYA YALÇIN, Bahar İPEK

**Affiliations:** 1 Department of Chemical Engineering, Faculty of Engineering, Middle East Technical University, Ankara Turkey

**Keywords:** Mesoporous zeolite, borosilicate, cetyltrimethyammonium bromide, ZSM-5

## Abstract

One-step facile synthesis of boron containing ZSM-5 microspheres is developed using 1,6-diaminohexane as the structure-directing agent and cetyltrimethylammonium bromide as the mesoporogen. High boron incorporation up to Si/B ratio of 38 is achieved and evidenced by the stretching vibrations of B–O–Si at 667 cm^-1^ and 917 cm^-1^ using Fourier-transform infrared spectra. The morphology of the crystals resembles berry-like spheres with sizes approximately 15 μm, which is composed of aggregated nanocrystals having sizes around 450 nm, is observed using scanning electron microscopy. The textural properties, i.e. the surface areas and pore volumes are investigated using N_2_ adsorption at –196 °C. t-plot micropore volume of 0.11 cm^3^/g and mesopore volume of 0.14 cm^3^/g are obtained applying this synthesis method for mesopores having pore diameters within the range of 2–10 nm.

## 1. Introduction

Zeolites are crystalline aluminosilicates composed of [SiO_4_] and [AlO_4_]^-^ tetrahedra that form uniform pores having pore sizes in the range of ~0.4 nm to 2 nm. They have been widely used in chemical industry as catalysts [1,2], ion-exchangers [3], and adsorbents [4]. Tetrahedral sites in zeolites can be isomorphously substituted by other atoms such as boron (B) [5], gallium (Ga) [5], titanium (Ti) [6], iron (Fe) [7] to be used as catalysts with improved activity and selectivity. Especially boron containing zeolites, also known as borosilicate or boralites [8], attracted a huge attention for various framework types. Incorporation of boron in many zeolite frameworks such as MFI [9], EUO [10], CHA [11], AFI [7], BEA [12], CON [13], FER [14], and more has been achieved.

Weaker acidity provided by boron-containing zeolites is intriguing for reactions that would require weak/mild acidity such as double-bond isomerization of linear olefins [15], methyl tertiary butyl ether (MTBE) cracking [16], Backmann rearrangement of cyclohexanone oxime [17–19] and cyclohexanol dehydration reaction [20]. Mild acidity provided by a combination of Al and B sites in zeolites is especially important for methanol to olefins (MTO) [9] and isomerization of styrene oxide to phenylacetaldehyde [21]. In these reactions, strong acidity (Brønsted acid, Al—OH sites) initiates the reaction and increases the conversion; however, it also results in deposition of carbonaceous aromatics such as polymethylbenzenes and polycyclic aromatics both inside the pores and the outer surface of the crystals [22]. In order to moderate the acidity, substitution of boron in the zeolite framework is used in addition to strategies such as promoter incorporation and metal doping [23]. Owing to the weak acid sites, boron-containing zeolites enhance catalytic stability and selectivity of products by decreasing the coke formation [9,24]. In addition to boron incorporation, adding mesopores to the structure (hierarchical ZSM-5) was reported to increase the lifetime of the catalyst due to the improved mass transfer rate [25].

Another reason for using borosilicates is to introduce new tetrahedral site atoms into the zeolite frameworks via post-synthetic techniques. Boron incorporated zeolites are potential intermediates for these zeolites because boron atoms can be extracted under mild acidic or thermal conditions [26]. Such deboronation and new tetrahedral substitution examples include large pore Ti containing zeolites (titanosilicates) for oxidation reactions [6,27] and [Fe]-SSZ-24 for isopropylation of biphenyl [7]. Easy deboronation of boron-containing zeolites are also useful to produce interconnected mesoporosity. Deboronation using steam treatment to create mesopores in the structure of [B]-zeolite Y are reported to provide higher catalytic activity in iso-octane and 1,3,5-triisopropylbenzene (TIPB) cracking reactions [28].

ZSM-5 is a highly versatile zeolite catalyst having MFI topology. Its inherent advantages such as 10-ring interconnected channel system (straight channels along the b axis (5.3 Å * 5.6 Å) and a sinusoidal channel along the a axis (5.1 Å * 5.5 Å)) and high Si/Al molar ratios make it a promising catalyst for many applications in refinery and petrochemical processes. Therefore, synthesis of ZSM-5 containing mesopores including nano-sized ZSM-5 [29] and hierarchical ZSM-5 [30] are frequently reported in the literature with the aim of increasing the lifetime of ZSM-5 catalysts.

Nanocrystalline zeolites are known to suffer from thermodynamic instability due to high surface energy and presence of large number of surface defects. Therefore, synthesis of hierarchical and microsized crystals are more sought for combined properties such as adequate acidity and easier diffusion rates. One way of synthesizing hierarchical zeolites is to simultaneously use microporogens (micropore structure-directing agents (SDA)) and mesoporogens in the synthesis gel. Use of an ordinary cationic surfactant such as cetyltrimethylammonium bromide (CTABr) as mesoporogen has been of interest since the discovery of mesoporous MCM series materials [31] due to its low cost and commercial availability. CTABr is a quaternary ammonium surfactant, which is frequently used in the synthesis of mesoporous M41S materials, which also directs microporous ZSM-5 synthesis in transition from mesoporous MCM-41 at elevated temperatures [32]. When CTABr is simultaneously used in the presence of a structure-directing agent such as tetraalkylammonium hydroxide to create mesopores in the zeolite framework, structure direction agent and CTABr compete with each other and amorphous mesoporous materials and bulky zeolites can be formed [33]. There are some ways to prevent this phase separation. For example, subnanocrystal-type zeolite seeds having a high degree of polymerization are firstly prepared by longterm aging and then CTABr is added to the system to prepare mesoporous zeolite so competition of CTABr with SDA decreases [34, 35].

 Another way to prevent the competition is that SDA can work after the formation of mesoporous structure. Firstly, mesoporous structures are formed using CTABr and it is occluded in mesopores to prevent growth of zeolite crystals. Then, SDA works to form zeolite structure [36]. 1, 6-diaminohexane (HDA) is an SDA used to synthesize ZSM-5. It is weaker than other SDAs. Using HDA, Chen et al. [37] synthesized nanosized ZSM-5 to create interparticle mesoporosity. However, mesopore volume was not high enough due to the fusion of nanosized particles. Xue et al. reported successful mesopore volumes of [Al]-ZSM-5 reaching 0.46 cm3 /g by combining HDA and CTABr as SDA and mesoporogen respectively [36]. In their method, simultaneous introduction of HDA and CTABr results in formation of mesoporous structures first, which is followed by crystallization of zeolite structures while CTABr is occluded in the mesopores to prevent growth and fusion of the zeolite nanoparticles.

In this work, mesoporous [B]-ZSM-5 zeolite synthesis was investigated using HDA as and cetyltrimethylammonium bromide (CTABr) as SDA and soft template, respectively. The effect of boron amount on crystallinity, morphology, surface area, and mesoporous volume was studied. This method enables high mesopore volume and high surface area without changing MFI topology while increased mesoporous volume and surface area are shown to be possible by increasing boron content.

## 2. Experimental procedure

### 2.1. Zeolite synthesis

Conventional [B]-ZSM-5 was synthesized hydrothermally for comparison following a procedure reported by Sanhoob et al. [38]. A gel mixture with the molar composition of 1 SiO_2_: 0.1 TPAOH: 0.1 NaOH: 0.1 H_3_BO_3_: 35.5 H_2_O was used. Firstly, 0.19 g of NaOH (Merck, 99 wt.%) was dissolved in 24.86 g of deionized water. Then, 2.44 g of TPAOH (Merck, 40 wt.% solution in water) as SDA was added and stirred for a few minutes until a homogeneous solution was obtained. After 7.2 g of colloidal silica (Sigma Aldrich, 40 wt.% suspension in water, LUDOX AS-40) was added to the solution dropwise under agitation, 0.296 g of boric acid (Merck, 99.5 wt.%, H_3_BO_3_) was added to the mixture. The mixture was stirred for 2 h at 25 °C. Then, it was transferred into 35-mL teflon-lined autoclaves and statically heated at 180 °C for 3 days. The product was recovered by vacuum filtration and washed with deionized water. Then, the solid was dried at 80 °C and calcined using muffle furnace at 550 °C for 6 h using a heating rate of 1 °C/min.

Conventional [Al]-ZSM-5 was synthesized following a procedure reported by Zhang et al. [39] with a gel formula of 1 SiO_2_: 0.0088 Al_2_O_3_: 0.255 Na_2_O: 0.256 TPABr: 182 H_2_O. Firstly, 0.05 g sodium aluminate (Reidel De Haen, 44 wt.% Na_2_O, 55 wt.% Al_2_O_3_, 1 wt.%H_2_O, NaAlO_2_) and 0.6 g of sodium hydroxide (Merck, 99 wt.%, NaOH) were dissolved in 101.25 mL H2 O and stirred for 12 h. After 6.425 g of tetraethyl orthosilicate (Merck, 98 wt.%, TEOS) was added dropwise under agitation, 2.1 g of tetrapropylammonium bromide (Merck, >99 wt.%, TPABr) as SDA was added and stirred for additional 12 h. Then, the mixture was transferred into 35-mL teflon-lined autoclaves and heated at 175 °C for 3 days. After that, the solid was separated by vacuum filtration, washed with distilled water several times, dried at 100 °C for 12 h and calcined at 550 °C using a heating rate of 1 °C/min for 5 h using muffle furnace.

Mesoporous B1-ZSM-5 was synthesized hydrothermally using a modified version of a procedure reported for mesoporous [Al]-ZSM-5 by Xue et al. [36]. Gel composition had the molar composition of 1 SiO_2_: x H_3_BO_3_: 0.13Na_2_O: 0.14 HDA: 0.1 CTABr: 60 H_2_O (x is 0.032 (B1-ZSM-5), and 0.128 (B2-ZSM-5)). For B1-ZSM-5, 1.04 g of NaOH (Merck, 99%) and 0.198 g of H_3_BO_3_ (boric acid, Merck, 99.5%) were dissolved in 108 g of distilled water. Then, 3.72 g of CTABr (Sigma Aldrich, 98%) and 1.66 g of HDA (Sigma Aldrich, 98%) were added and dissolved. And then 6.01 g of fumed silica (Sigma Aldrich, 99.9%) was added. After obtained mixtures were stirred for 6 h at 25 °C, they were transferred into teflon-lined autoclaves and hydrothermal synthesis was performed at 150 °C for 14 days. The products were recovered by vacuum filtration and washed with 250 mL deionized water. Later, they were dried in oven at 80 °C overnight and calcined under flowing air (40 cm^3^/min flow rate) at 580 °C for 10 h using a heating rate of 1 °C/min. The same procedure was applied for synthesis of B2-ZSM-5 using a boric acid molar ratio of 1 SiO_2_: 0.128 H_3_BO_3_.

### 2.2. Characterization

Powder X-ray diffraction data were recorded on Rigaku Ultima-IV diffractometer using Cu Kα source (λ = 1.5418 Å) operated at 40 kV and 30 mA, using 2? angle range from 2°to 50°at a speed of 1 °/min. Measured diffractograms were analyzed using CelRef Unit-Cell refinement software1Laugier J, Bochu B. (2019) CELREF Unit-Cell refienement software [online]. Website http://www.ccp14.ac.uk/tutorial/lmgp/celref.htm. [accessed 01 September 2019], where unit cell parameters of an orthorhombic unit cell system with a space group of *Pnma* were refined for calcined samples.

Scanning electron microscopy (SEM) images were obtained using a QUANTA 400F Field Emission SEM with an accelerating voltage of 30 kV. The boron and silicon content of the catalyst samples were analyzed using inductively coupled plasma-optical emission spectrometer (ICP-OES, PerkinElmer Optime 4300DV).

Surface area and pore volume of the calcined zeolites were analyzed using Micromeritics Tristar II 3020 analyzer. Samples were degassed at 300 °C for 6 h under vacuum (using Micromeritics VacPrep 061) before N_2_ adsorption/desorption tests were conducted at –196 °C. N_2_ (Oksan, 99.999%) adsorption tests were conducted at relative pressure values between 10^-5^ and 0.98 following the free cell volume measurement conducted using He (Oksan, 99.999%). Micropore volumes were calculated using t-plot statistical thickness method between 3.5 Å and 5 Å with Harkins Jura thickness equation [40]. The pore size distributions were calculated using Barrett–Joyner–Halenda (BJH) Adsorption model [41] and nonlocal density functional theory (NLDFT) with cylinder pore size assumption from adsorption branch.

Fourier-transform infrared spectra (FTIR) was obtained using PerkinElmer UATR Two spectrometer equipped with an Attenuated Total Reflectance (ATR) attachment. The samples were scanned 64 times at a spectral resolution of 4 cm^-1^. The spectrum was collected in the mid IR region from 500 cm^-1^ to 4000 cm^-1^.

Thermal gravimetric analysis (TGA) of as-prepared samples were performed using a thermal analyzer (Shimadzu, DTG 60H) from 30 C to 800 °C using 5 °C/min heating rate and 60 cm^3^/min air flow.

## 3. Results and discussion

### 3.1. Crystallinity and Boron Incorporation

**Figure 1:**
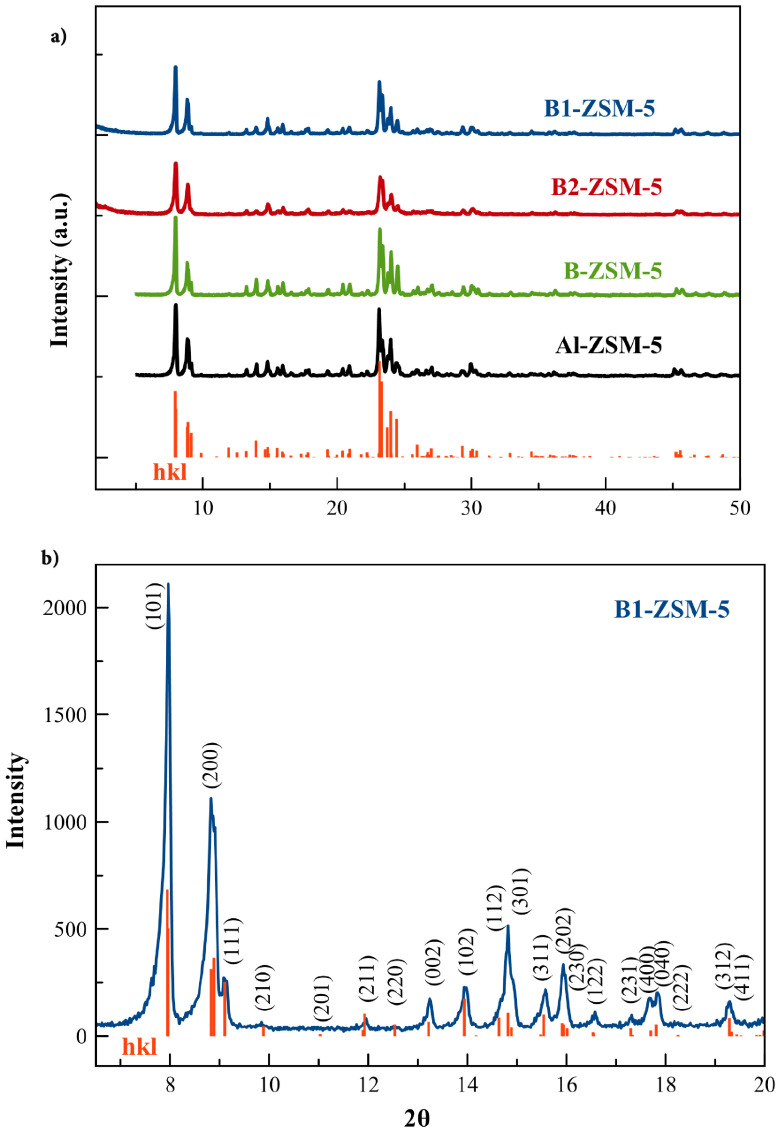
a) XRD patterns of mesoporous B1-ZSM-5, mesoporous B2-ZSM-5, conventional B-ZSM-5 and Al-ZSM-5 (λ = 1.5418 Å) and expected pattern for ZSM-5 retrieved from International Zeolite Association, Database of Structures (http://www.iza-structure.org/databases/). b) XRD pattern of mesoporous B1-ZSM-5, Miller indices (hkl) and corresponding 2θ positions for ZSM-5, λ = 1.5418 Å, orthorhombic unit cell Pnma space group and a = 20.022 Å, b = 19.899 Å, c = 13.383 Å.

Powder XRD pattern of the mesoporous [B]-ZSM-5 zeolites and conventional B-ZSM-5 are shown in Figure 1a. All samples show typical MFI pattern with high crystallinity. No extra phase was observed within the sensitivity of the laboratory scale X-ray diffractometer (Rigaku Ultima-IV diffractometer, see Figure 1b). The addition of CTABr and incorporation of boron did not affect the crystallinity of the synthesized zeolites. According to Xue et al., the optimum CTABr/SiO_2_ ratio that shows optimum mesopore and micropore formation is 0.1 for [Al]-ZSM-5 synthesis. Larger CTABr/SiO_2_ ratios such as 0.2 are reported to cause poor crystallinity due to the hindered nucleation and crystal growth due to the long hydrophobic carbon chains of CTABr [36]. The CTABr/SiO_2_ ratio of 0.1 used here is shown to result in zeolite crystal growth with larger crystal sizes in B1-ZSM-5 when compared to B2-ZSM-5.

**Figure 2:**
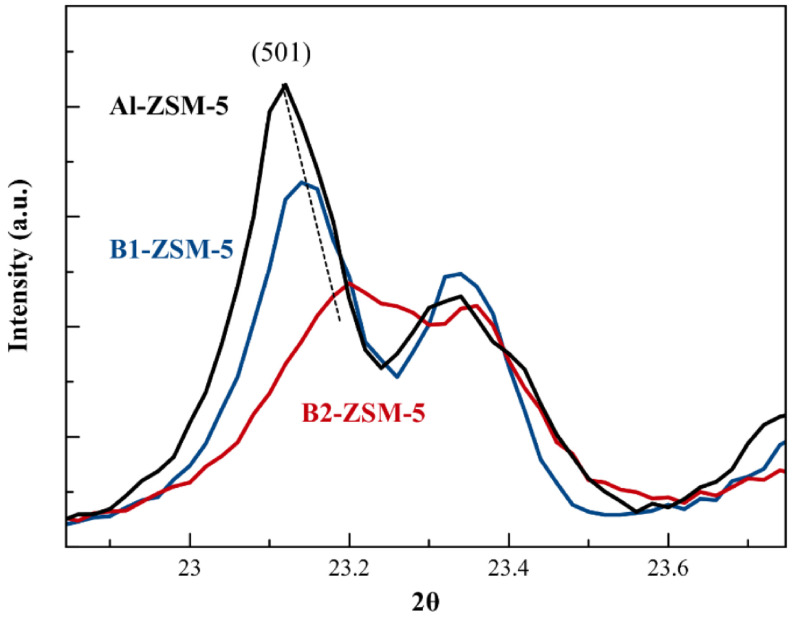
XRD patterns of mesoporous B1-ZSM-5 and B2-ZSM-5 and conventional Al-ZSM-5 at 2θ angles between 22°and 24°(λ = 1.5418 Å).

The boron incorporation into the framework can be checked by investigating the unit cell parameters of the samples. Since B–O bonds in the zeolite framework are much shorter (1.39 Å [42]) than Si–O (1.61 Å [43]) or Al–O (1.75 Å [44]) bonds, the unit cell volume of the B-containing samples are expected to have a lower value when compared to [Al]-ZSM-5. As it can also be understood from the increasing 2θ angle belonging to (501) plane of *Pnma* space group, (23.12°for Al-ZSM-5, 23.14°for B1-ZSM-5 and 23.20°for B2-ZSM-5, see Figure 2), the unit cell volume of boron containing ZSM-5 shows decreased values with increasing boron content in the framework. The refined unit cell parameters for ZSM-5 samples having a *Pnma* space group are given in Table 1.

**Table 1:** Refined unit cell parameters for calcined ZSM-5 using CelRef unit-cell refinement software.

Sample	a/ Å	b/ Å	c/ Å	V/ Å3
B1-ZSM-5	20.012	19.860	13.370	5313
B2-ZSM-5	20.014	19.814	13.358	5297
Al-ZSM-5	20.083	19.871	13.355	5330

**Figure 3:**
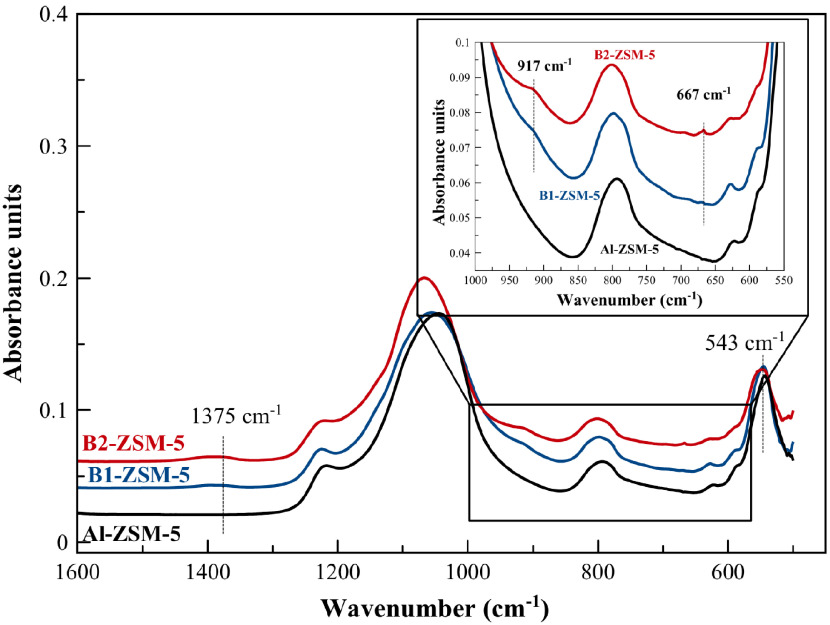
FTIR spectra of hydrated B1- ZSM-5, B2-ZSM-5, and Al-ZSM-5.

FTIR spectra of the hydrated samples show absorption bands of lattice vibrational modes at 1216 cm^-1^, 1044 cm^-1^ (external and internal asymmetric TOT stretch), 800 cm^-1^ (external symmetric TOT stretch), 627 cm^-1^, 587 cm^-1^, and 543 cm^-1^ (Figure 3). The absorption band observed at 543 cm^-1^ is ascribed to the presence of double five membered ring vibrations, indicating formation of pentasil zeolite structure [45] in B1-ZSM-5 and B2-ZSM-5.

In addition to the lattice vibrational modes observed both on Al-ZSM-5 and B-ZSM-5, out-of-plane bending and stretching vibrations of B–O–Si (tetrahedrally coordinated B) at 667 cm^-1^ and 917 cm^-1^ [42] were additionally observed on B1-ZSM-5 and B2-ZSM-5, indicating boron incorporation to the MFI framework. The intensity of the absorption bands at 667 cm^-1^ and 917 cm^-1^ were observed to increase with increasing boron content. The vibration at 1375 cm^-1^ shows presence of trigonal B species (asymmetric B–O stretching) in the framework [42], which is commonly observed when the B-containing zeolites are not fully hydrated [46].

Table 2 gives the elemental composition of the synthesized zeolites. Si/B ratios of the zeolites were obtained from ICP-EOS method following calcination of the samples. Si/B ratios of all resulting zeolites are lower than those of the synthesis gel (Si/B= 31 & 8 respectively in B1- and B2-ZSM-5 in synthesis gel). Boron content of synthesized zeolites increases with increasing boron content in the synthesis gel. Boron content in B2-ZSM-5 is found to be higher than B1-ZSM-5 as expected (Si/B of 38 vs. 78), and also evidenced from the XRD patterns (Figure 2). Relatively high boron content of B1-ZSM-5 is beneficial for this zeolite to be further used in isomorphic substitution of boron with other tetrahedral atoms.

**Table 2:** Elemental analysis of the microporous/mesoporous [B]-ZSM-5.

Samples	Si/B or Si/Al in gel mixture	Si/B or Si/Al (a)
B1-ZSM-5	31	78
B2-ZSM-5	8	38
Conventional B-ZSM-5	10	55
Conventional Al-ZSM-5	56	43±3

a: Si/B from ICP-OES, Si/Al from EDX analysis

The maximum B content of microporous [B]-ZSM-5 sample was reported to have a Si/B ratio of 36 (~2.6 B/unit cell) for a synthesis gel mixture composition of SiO_2_:B_2_O_3_ = 10:1 and SiO_2_:Na_2_O_3_ = 10.5:1 [47]. The resulting boron content of the microporous zeolite was improved by decreasing the SiO2 :Na2 O3 ratio to 3.5:1 [47]. As the pH of the synthesis gel decreases with increasing H_3_BO_3_, the addition of NaOH becomes vital that accelerates the dissolution of boron source and incorporation of B into the zeolite framework [8]. Hence, the highest achieved Si/B ratio of 38 here, could be further improved by increasing the NaOH content in the synthesis gel from SiO_2_:Na_2_O_3_ of 1:0.13 (or SiO_2_:Na_2_O_3_ of 7.7:1) to 1:0.28.

### 3.2. Morphology

**Figure 4:**
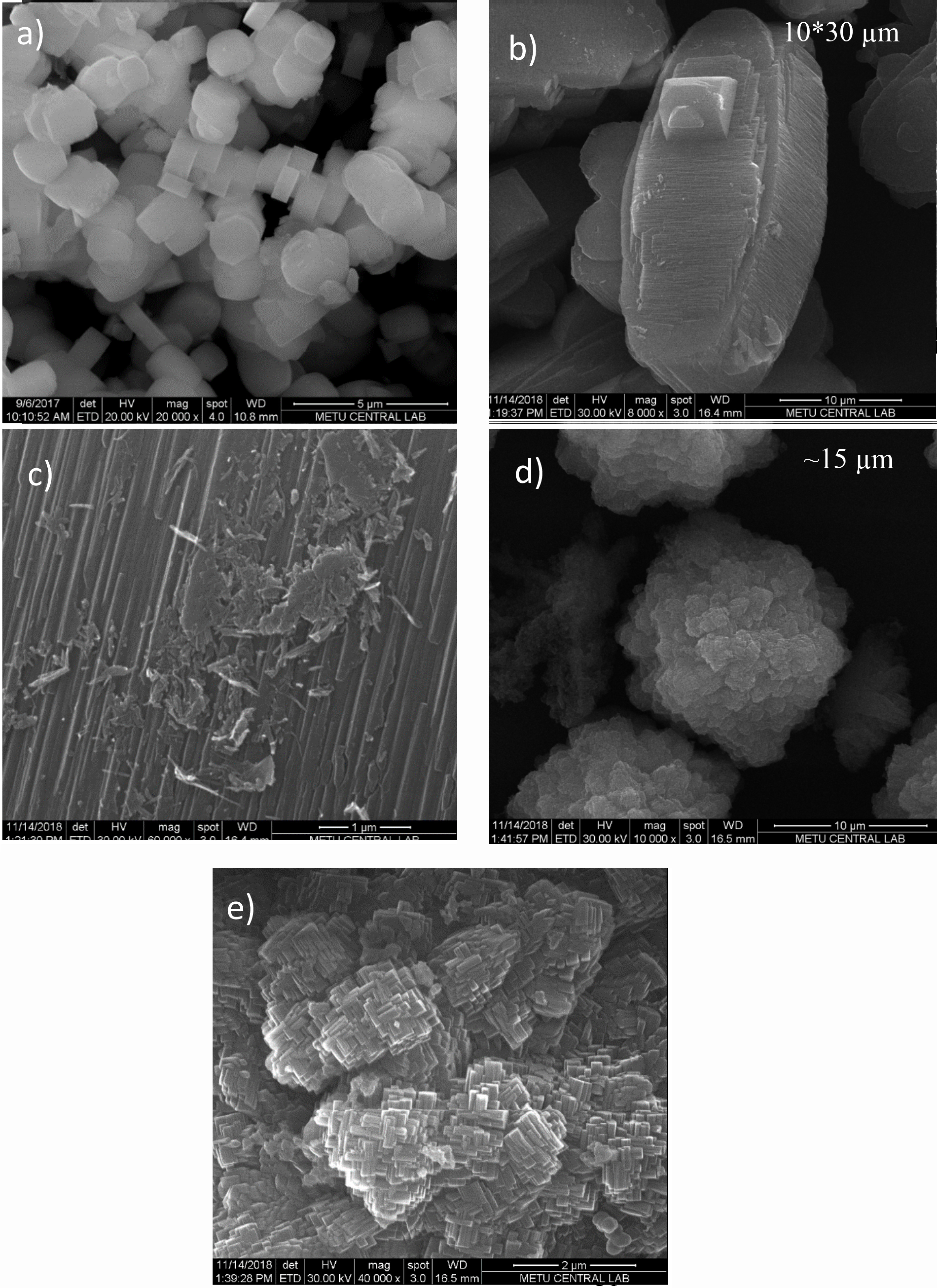
SEM images of conventional B-ZSM-5 (a), B1-ZSM-5 (b, c), and B2-ZSM-5 (d, e).

SEM images of the synthesized and calcined samples are shown in Figure 4. Conventional B-ZSM-5 shows typical coffin-shaped crystal morphology with particle sizes between 1 and 2 μm (Figure 4a). Mesoporous B1-ZSM-5 and B2-ZSM-5 showed different particle sizes with varying boron content. Lower boron content in the gel mixture (B1-ZSM-5) resulted in large crystals approximately 30 ×10 μm with layers observed at the crystal surface (see Figures 4b and 4c). B2-ZSM-5, having a higher boron content in the gel mixture resulted in berry-like clusters having sizes around 15 ×20 μm, composed of nanocrystals having sizes approximately 450 nm (Figures 4d and 4e). Figures 4c and 4e represent B1-ZSM-5 and B2-ZSM-5 at higher magnification. Nanoparticle structure of B2-ZSM-5 creates intercrystalline mesoporosity (Table 3). On the other hand, surface of B1-ZSM-5 was layer-like and nanoparticles were not observed.

**Table 3:** Textural characteristic results of the microporous/mesoporus [B]-ZSM-5.

Samples	S_BET_ (m^2^/g)	S^(a)^_mic_ (m^2^/g)	S_EXT_ (m^2^/g)	S_EXT_/S_BET_	V^(b)^_total_ (cm^3^/g)	t-plot V^(c)^_micro_ (cm^3^/g)	V^(d)^_meso_ (cm^3^/g)	V_meso_/V_total_
B1-ZSM-5	351	199	152	0.43	0.21	0.10	0.11	0.53
B2-ZSM-5	342	117	225	0.66	0.25	0.11	0.14	0.56
Conventional B-ZSM-5	346	256	90	0.26	0.18	0.13	0.05	0.29
Conventional Al-ZSM-5	342	286	56	0.16	0.16	0.14	0.02	0.11

a: S_mic_ = t-plot surface area, S_EXT_ = S_BET_-S_mic_ b: V_total_ = Total pore volume determined from the adsorbed amount at P/Po= 0.984 c: t-plot V_micro_ = Micropore volume calculated using t-plot method d: V_mesa_ = V_total_- t-plot V_micro_

The aggregation of nanoparticles to microspheres were reported when HDA was used as the SDA with [36] or without the presence of CTABr [37]. As Xue et al. reported, presence of CTABr is known to increase the sizes of the microspheres. They reported microsphere sizes around 9 μm for [Al]-ZSM-5 following a 14-day synthesis period [36]. In our case, B2-ZSM-5 sample having a relatively higher B content (Si/B = 38, see Table 2) resulted in clusters having sizes approximately 15 μm following a 14-day synthesis procedure with the same CTABr/SiO_2_, Na_2_O/SiO_2_, and H_2_O/SiO_2_ ratios when compared to [Al]-ZSM-5 reported by Xue et al. [36]. B1-ZSM-5, on the other hand, having a Si/B ratio of 78, resulted in larger crystals with different morphology. Similar large microporous zeolite crystal growth was also reported for Si/Al ratios reaching 200 by Xue et al., and explained by enhanced nucleation and growth rates in presence of higher Si content [36]. This enhanced crystallization rates disrupted the comparable rates of mesopore formation and zeolite crystallization, which led to lower mesopore volumes (see Table 3).

### 3.3. Textural property characterizations

**Figure 5:**
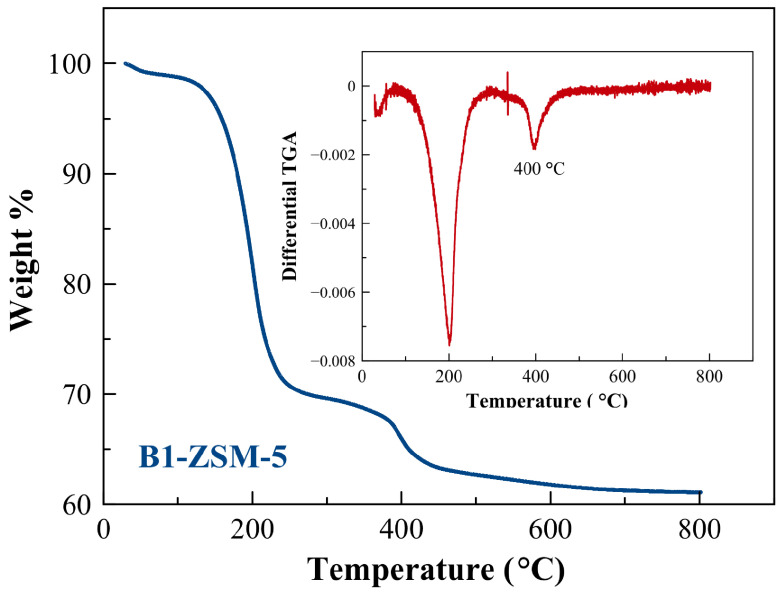
TGA and differential TGA of as-prepared B1-ZSM-5.

**Figure 6:**
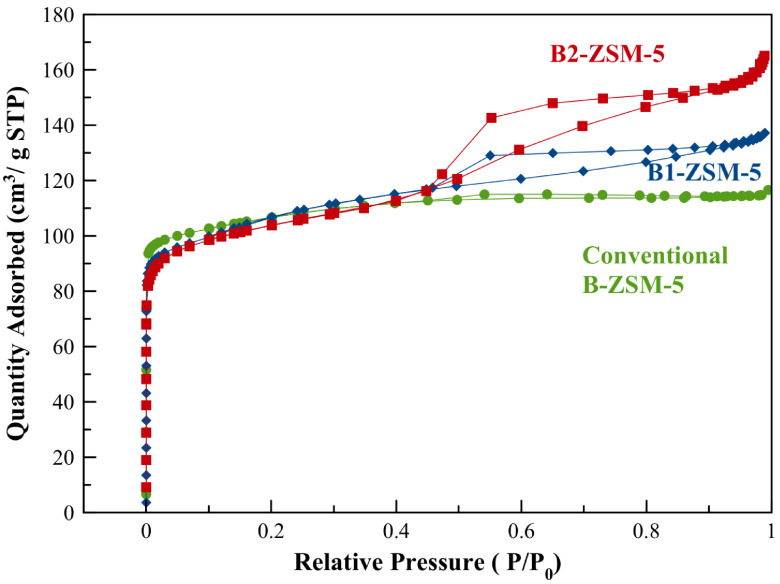
N_2_ adsorption–desorption isotherm of mesoporous B1-ZSM-5 and B2-ZSM-5 and conventional B-ZSM-5 obtained at –196 °C.

TGA of as-prepared B1-ZSM-5 was performed prior to any textural analysis via N2 adsorption to make sure total removal of the organics used in synthesis. Based on the TGA and the differential TGA results (Figure 5), the calcination temperature was selected as 580 °C, which was maintained for 10 h for complete combustion of the organics. Figure 6 represents the N2 adsorption-desorption isotherms obtained at –196 °C following the calcination of the samples at 580 °C. According to Figure 6, addition of CTABr resulted in increased N2 adsorption capacity and hysteresis. Conventional B-ZSM-5 exhibited a typical type-I isotherm according to IUPAC classification, reflecting traditional zeolite having only micropores. However, B1-ZSM-5 and B2-ZSM-5 showed type-IV isotherms with a significant hysteresis loop in the P/P0 range from 0.45 to 0.9, indicating mesoporosity. Increasing amount of boron content increased the adsorption capacity and hysteresis. The hysteresis type can be designated as H2(b), indicating pore-blocking with a wider range of pore necks [48].

Table 3 gives the textural characterization of the synthesized zeolites using N2 adsorption isotherms. The microporous surface area was calculated using t-plot method, whereas BET surface area was calculated assuming multilayer adsorption model. The external surface area was calculated as the difference between the BET surface area and the microporous surface area. B2-ZSM-5, with the highest boron incorporation into the framework resulted in larger external surface areas and larger S_EXT_/S_BET_ ratios when compared to B1-ZSM-5 and conventional B-ZSM-5.

The micropore volumes of mesoporous B-ZSM-5 were calculated using statistical thickness method (tplot) for all samples, which showed slight decreases compared to conventional B-ZSM-5 (micropore volume of B1-ZSM-5 and B2-ZSM-5 are 0.10 and 0.11 cm^3^/g, respectively, see Table 3). The mesopore volume values were calculated by subtracting the t-plot micropore volume from the single point pore volume obtained at P/P_0_ = 0.98. As it can be inferred from Table 3, increasing amount of boron content increased the mesopore volume of the synthesized zeolites. The highest mesopore volume was obtained on B2-ZSM-5, which was 0.14 cm^3^/g. B1-ZSM-5 showed a mesopore volume of 0.11 cm^3^/g.

Reported mesopore volume values for [Al]-ZSM-5 with the same CTABr/SiO_2_, Na_2_O/SiO_2_, and H_2_O/SiO_2_ ratios but higher Al incorporation in the framework (Si/Al = 30) were higher than the B2-ZSM-5 sample here. [Al]-ZSM-5 resulted in 0.32 cm^3^/g of mesopore volume, whereas B2-ZSM-5 resulted in 0.14 cm^3^/g, which could be due to lower interaction of boron content with CTABr when compared to Al sites.

**Figure 7:**
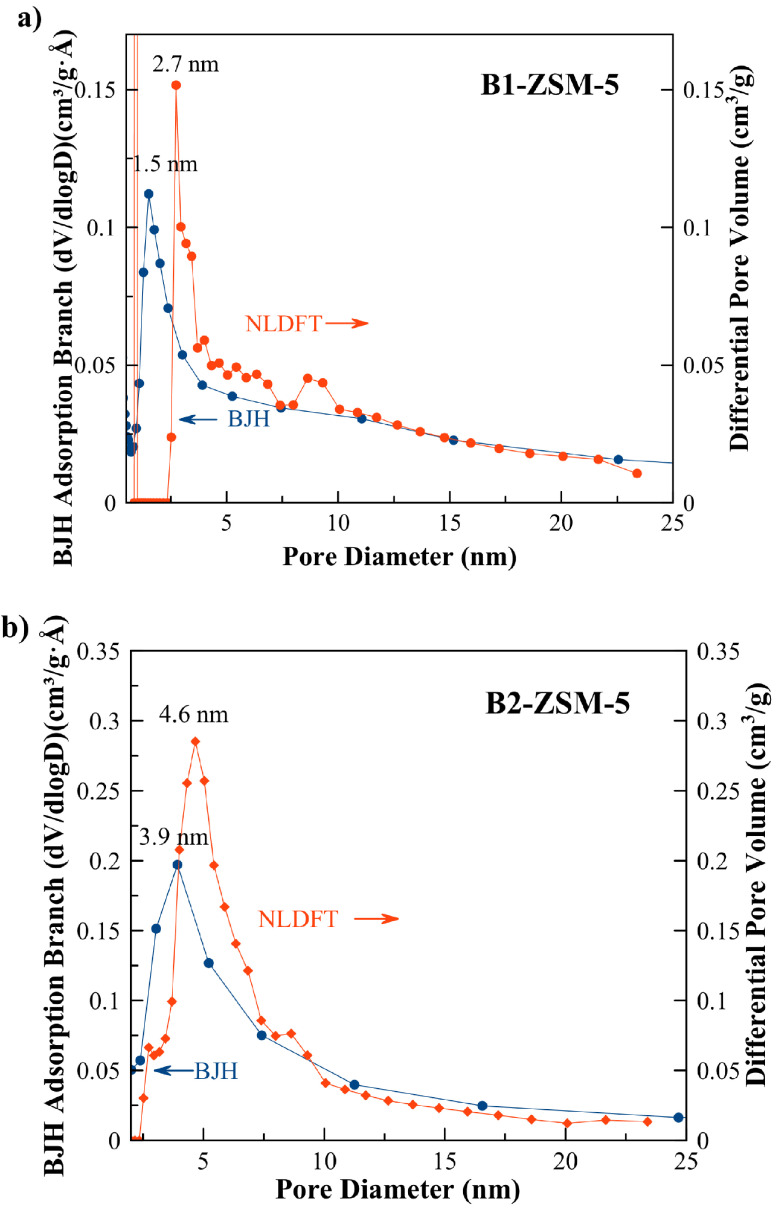
BJH adsorption branch and NLDFT pore size distribution of a) mesoporous B1-ZSM-5 and b) B2-ZSM-5.

Pore sizes of the samples were calculated using Barrett–Joyner–Halenda (BJH) adsorption [41] and NLDFT pore size methods (see Figures 7a and 7b). According to BJH adsorption method B1-ZSM-5 had small pores which are ~1.5 nm, (it showed ~2.7 nm pores extending to 20 nm with NLDFT pore size method). B2-ZSM-5 had larger pores than B1-ZSM-5. According to BJH adsorption and NLDFT pore size methods, B2-ZSM-5 showed pore sizes in the range of ~3.9 nm and ~4.6–10 nm, respectively.

## 4. Formation mechanism

**Figure 8:**
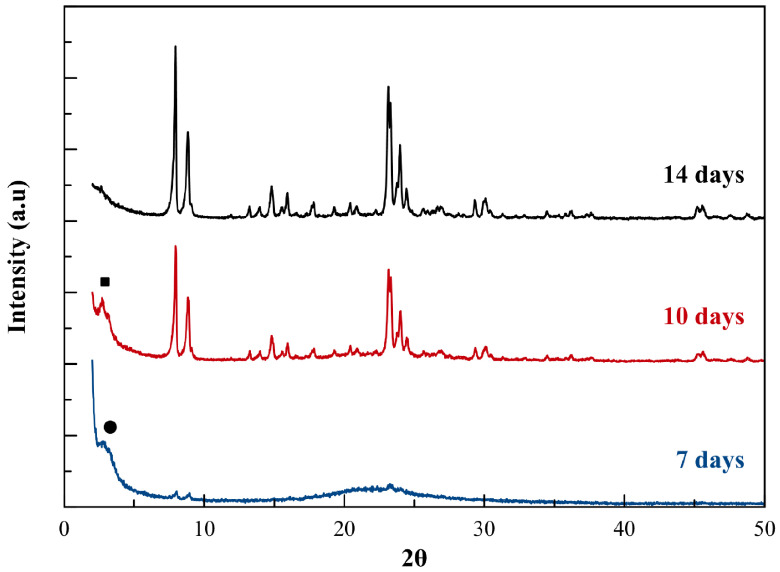
XRD pattern of mesoporous B2-ZSM-5 following the 7th, 10th, and 14th day of synthesis (λ = 1.5418 Å) •: peak at 2.92° ■ : peak at 2.72°.

We investigated the formation mechanism of the microspheres of mesoporous B2-ZSM-5 using XRD, SEM, and N2 adsorption analysis of calcined samples at the 7th, 10th and 14th day of synthesis (the synthesis of B2-ZSM-5 is repeated for this part). At the 7th day of synthesis, the peaks characteristic of MFI phase (with the most observable peaks at 8.02°and 8.94°belonging to the (101) and (020) planes of *Pnma* space group) appeared at low intensity values, indicating the starting of the crystallization (see Figure 8). In addition to the MFI peaks, a broad peak at 2θ value of 2.92°corresponding to a d-spacing of 3 nm was observed together with a broad peak between 20°and 25°indicating a mesoporous structure. At the 10th day of synthesis, the peak at 2.92°shifted to 2.72°(d-spacing of 3.2 nm), and the characteristic MFI peaks gained more intensity. At the 14th day of synthesis, the peak at 2.72°and the broad peak between 20°and 25°disappeared as the MFI peaks reached their maximum intensity. When the intensity of the peak belonging to the (101) plane of ZSM-5 ( *Pnma* space group) observed at 7.94°was compared at the 10th day and 14th day of the synthesis, it can be said that the sample at the 10th day has 68% crystallinity with respect to the one at the 14th day.

**Figure 9:**
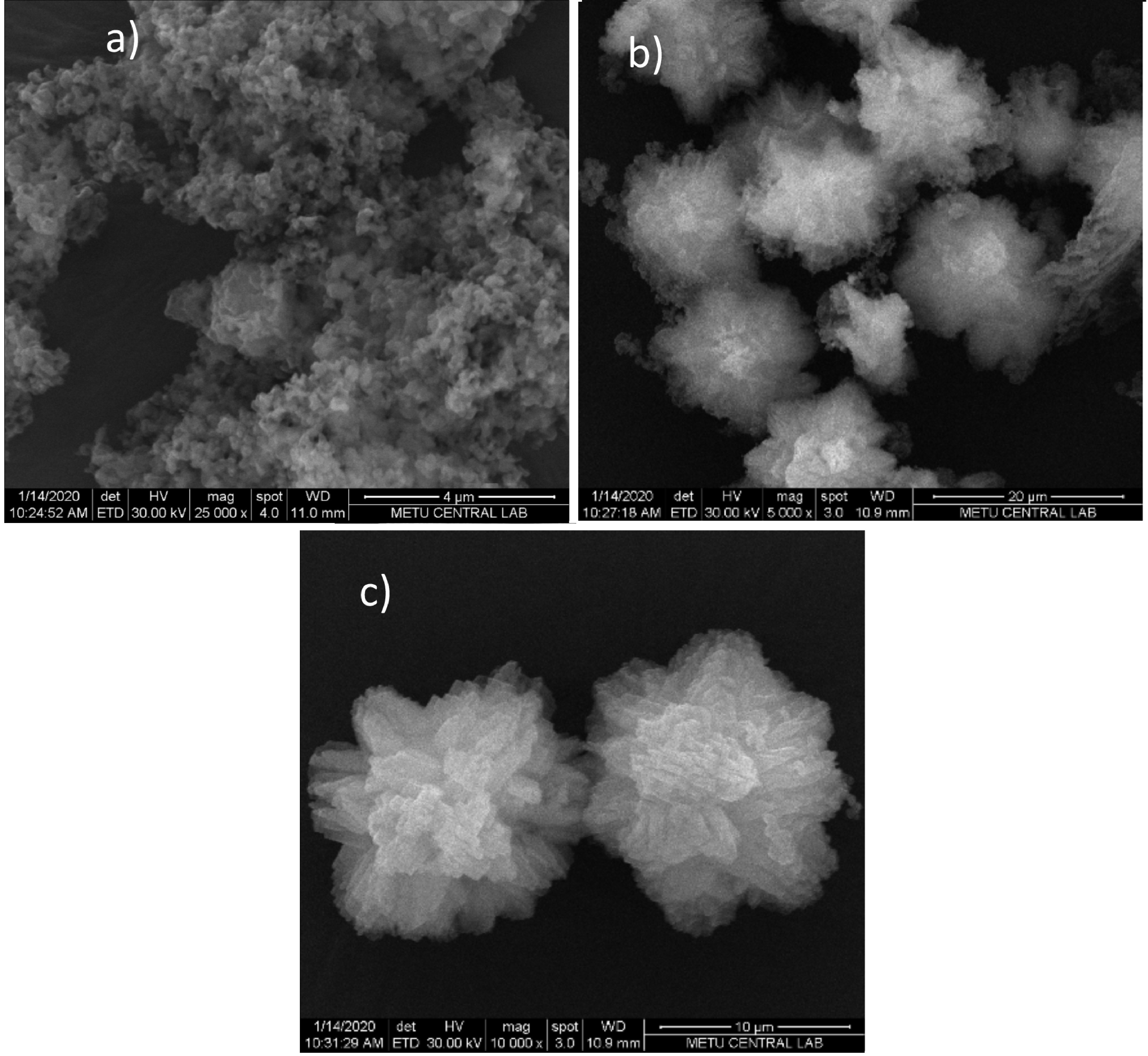
SEM images of B2-ZSM-5 at the 7th day (a), 10th day (b), and 14th day (c) of synthesis.

**Figure 10:**
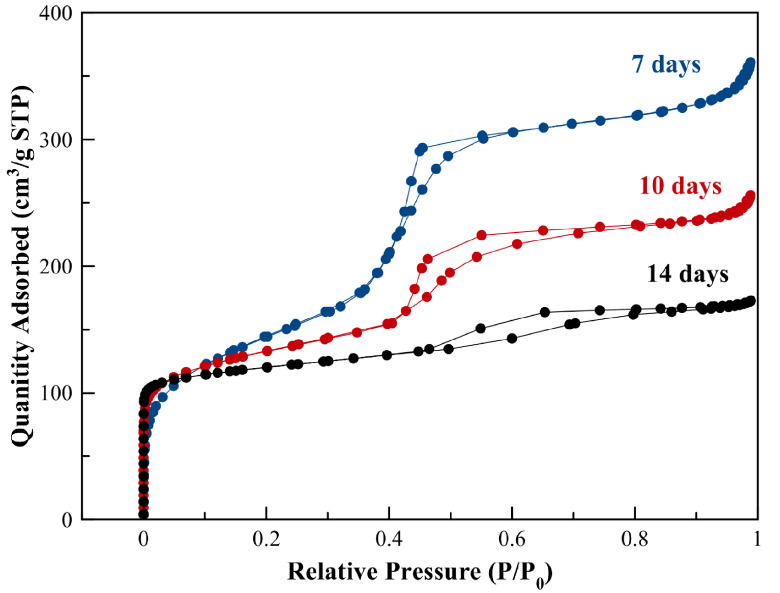
N_2_ adsorption–desorption isotherm of mesoporous B2-ZSM-5 at the 7th, 10th and 14th day of hydrothermal synthesis.

**Figure 11:**
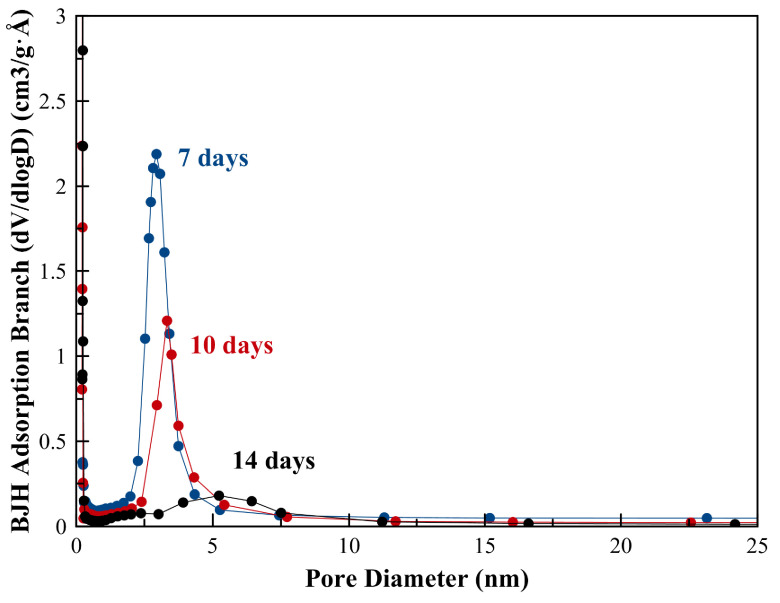
BJH adsorption branch pore size distribution of mesoporous B2-ZSM-5.

SEM images (see Figures 9a–9c) show that aggregates of spherical particles having sizes approximately 200 nm (see Figure 9a) occur at the 7th day of the synthesis that are mesoporous (see Table 4 for mesopore volume and Figure 10) as also indicated from XRD pattern. The micropore formation was negligible (0.01 cm^3^/g) when compared to mesopore volume found by N_2_ adsorption at –196 °C (0.54 cm^3^/g, Table 4). The pore size distribution of this sample showed a fairly narrow pore size range with a maximum value of 2.9 nm (see Figure 11), which is in agreement with the d-spacing value found in XRD analysis. The morphology and the narrow pore size distribution at around 3 nm found for this mesoporous sample resemble those of an MCM-41 structure [1].

**Table 4:** Textural characteristic results of the B2-ZSM-5 with respect to synthesis time.

Time (day)	S_BET_ (m^2^/g)	S^(a)^_mic_ (m^2^/g)	S_EXT_ (m^2^/g)	S_EXT_/S_BET_	V^(b)^_total_ (cm^3^/g)	t-plot V^(c)^_micro_ (cm^3^/g)	V^(d)^_mesa_ (cm^3^/g)	V_meso_/V_total_
7	508	18	490	0.96	0.55	0.01	0.54	0.98
10	448	189	259	0.58	0.39	0.09	0.30	0.77
14	395	268	127	0.32	0.27	0.13	0.14	0.52

a: S_mic_ = t-plot surface area, S_EXT_ = S_BET_ -S_mic_ b: V_total_ = Total pore volume determined from the adsorbed amount at P/Po= 0.984 c: t-plot V_micro_ = Micropore volume calculated using t-plot method d: V_meso_ = V_total_- t-plot V_micro_

This mesoporous spherical structure enlarges to diameters of ~13–15 μm (see Figure 9b) while some part of it converts into microporous zeolite at the 10th day of the synthesis. The micropore volume obtained at the 10th day is 0.09 cm^3^/g (see Table 4) while the mesopore volume value dropped to 0.30 cm^3^/g. Then, further crystallization under the effect of HDA gives more defined crystals at the 14th day (see Figure 9c) resulting in a higher micropore volume (see Table 4, 0.13 cm^3^/g) and a slightly lower mesopore volume (0.14 cm^3^/g).

The trends in the micropore–mesopore volume changes during the synthesis confirm a sequential mesostructure formation and crystallization of mesoporous structure to zeolite. Xue et al. also reported formation of mesoporous materials (nanoparticles similar to Al-MCM-41) in the presence of CTABr, followed by conversion of the mesostructured material to the crystalline ZSM-5 starting from the 9th day of synthesis using XRD, FTIR, and N_2_ adsorption [36]. They explained this sequential mechanism by relatively weak interaction of the structure-directing agent: HDA with the oligomers, which prevented the competition between CTABr and SDA (and hence prevented the formation of a mixture of amorphous mesostructure and zeolite crystals) [36]. A similar mechanism is suggested here for mesoporous B2-ZSM-5 with slight changes in the resulting mesoporous structure observed at the 7th day of the synthesis. The mesopore volumes of B2-ZSM-5 are found to be smaller (0.54 cm^3^/ g) than mesoporous Al-ZSM-5 reported by Xue et al. (0.89 cm^3^/g mesopore volume at the 7th day of [Al]-ZSM-5 [36]). The reason for this can be the lower B concentration incorporated into the framework unlike high Al content of Al-ZSM-5. The weaker interaction of B and CTABr could result in slightly smaller mesopore volumes. Furthermore, the hysteresis observed in Figure 10 for the 7th day synthesis can be classified as the H2 type [48], which is attributed to blocked pores unlike type H1, attributed to open pores typical for MCM-41. Nevertheless, formation of aggregated spherical mesoporous particles of sizes approximately 200 nm at the beginning of the synthesis is still suggested here.

The pore diameter distribution in B2-ZSM-5 was also investigated at the 7th, 10^th^, and 14th day of the hydrothermal synthesis. The pore sizes get wider from ~3 nm to ~5–10 nm as the conversion from total mesopore structure to small zeolite crystal aggregates takes place (see Figure 11). The pore size of 2.9 nm and 3.3 nm observed at the 7th and 10th day of synthesis are in very good agreement with the d-spacing values of 3 and 3.2 nm calculated from XRD (Figure 8). A similar pore size distribution is also reported for mesoporous Al-ZSM-5 by Xue et al. The pore diameter of the mesostructures was observed approximately 3 nm, whereas with progressed crystallization, pore size distribution becomes wider and shifts to larger pore diameters ~12 nm [36].

## 5. Conclusion

One-step synthesis of hierarchical borosilicate [B]-ZSM-5 has been shown for Si/B ratios ranging from 38 to 78 using 1,6-diaminohexane as the structure-directing agent and cetyltrimethylammonium bromide as the mesoporogen. The morphology of the samples resembled microspheres with particle sizes varying between 10 and 30 μm formed by aggregation of nanoparticle crystals. The microspherical form was related to the used structure agent, i.e. 1,6-diaminohexane, whereas the nanoparticles having sizes approximately 450 nm were related to the presence of cetyltrimethylammonium bromide acting as the crystal growth inhibitor. Mesopore volumes reaching 0.14 cm^3^/g was related to the interaction of boron with cetyltrimethylammonium bromide due to the increasing behavior of mesopore volumes with increasing boron content. The formation mechanism of the microspheres is shown to start with amorphous and mesoporous particles, which then crystallizes into ZSM-5 with the effect of 1,6-diaminohexane.

## References

[1] (1997). From microporous to mesoporous molecular sieve materials and their use in catalysis. Chemical Reviews.

[2] (2016). Catalytic test reactions for the evaluation of hierarchical zeolites. Chemical Society Reviews.

[3] (2001). Ion exchange in zeolites. Studies in Surface Science and Catalysis. Elsevier.

[4] (2009). Intermolecular Forces in Zeolite Adsorption and Catalysis. Ordered Porous Solids: Recent Advances and Prospects.

[5] (2013). Simultaneous isomorphous incorporation of boron and germanium in MFI zeolites. Microporous and Mesoporous Materials.

[6] (2015). Ccaron;ejka J. Titanium impregnated borosilicate zeolites for epoxidation catalysis. Microporous and Mesoporous Materials.

[7] (2011). -SSZ-24 through the isomorphous substitution of [B]-SSZ-24 with iron, and its catalytic properties in the isopropylation of biphenyl. Journal of Molecular Catalysis A: Chemical.

[8] (1999). Synthesis and characterization of boron-containing molecular sieves. Topics in Catalysis.

[9] (2014). Highly stable boron-modified hierarchical nanocrystalline ZSM-5 zeolite for the methanol to propylene reaction. Catalysis Science and Technology.

[10] (2001). Synthesis and characterization of borosilicates with the EUO framework topology. Microporous and Mesoporous Materials.

[11] (2007). Effect of boron substitution in chabazite framework: IR studies on the acidity properties and reactivity towards methanol. Journal of Physical Chemistry C.

[12] (2012). Control of Al for B framework substitution in zeolite Beta by counterions. Microporous and Mesoporous Materials.

[13] (2014). \v{Z}ilkov&aacute; N. Catalytic applications and FTIR investigation of zeolite SSZ-33 after isomorphous substitution. Microporous and Mesoporous Materials.

[14] (2002). B-containing molecular sieves crystallized in the presence of ethylenediamine. Part I: crystal structure of as-synthesized B-FER Giovanni. Microporous and Mesoporous Materials.

[15] (1986). Developments in Zeolite Science and Technology.

[16] (2024790).

[17] (1996). Vapour-phase Beckmann rearrangement using B-MFI zeolites. Applied Catalysis A: General.

[18] (2000). Active sites of a [B]-ZSM-5 zeolite catalyst for the Beckmann rearrangement of cyclohexanone oxime to caprolactam. Journal of Catalysis.

[19] (2010). Modelling active sites for the Beckmann rearrangement reaction in boron-containing zeolites and their interaction with probe molecules. Physical Chemistry Chemical Physics.

[20] (1994). Catalytic conversion of oxygen containing cyclic compounds. Part I. Cyclohexanol conversion over H[Al]ZSM-5 and H[B]ZSM-5. Journal of Molecular Catalysis.

[21] (2014). Catalytic performance of boron and aluminium incorporated ZSM-5 zeolites for isomerization of styrene oxide to phenylacetaldehyde. Microporous and Mesoporous Materials.

[22] (2015). Coke formation and deactivation pathways on H-ZSM-5 in the conversion of methanol to olefins. Journal of Catalysis.

[23] (2009). Louis B. Journal of Catalysis.

[24] (2015). Effect of boron incorporation on the structure, products selectivities and lifetime of H-ZSM-5 nanocatalyst designed for application in methanol-to-olefins (MTO) reaction. Microporous and Mesoporous Materials.

[25] (2008). Selective production of propylene from methanol: Mesoporosity development in high silica HZSM-5. Journal of Catalysis.

[26] (1994). Synthesis and characterization of pure-silica and boron-substituted SSZ-24 using N(16) methylsparteinium bromide as structure-directing agent. Microporous Materials.

[27] (2019). Synthesis of Ti-containing extra-large-pore zeolites of Ti-CIT-5 and Ti-SSZ-53 and their catalytic applications. Microporous and Mesoporous Materials.

[28] (2017). Catalytic function of boron to creating interconnected mesoporosity in microporous Y zeolites and its high performance in hydrocarbon cracking. Journal of Catalysis.

[29] (2014). Direct synthesis of hierarchical ZSM-5 zeolite and its performance in catalyzing methanol to gasoline conversion. Industrial and Engineering Chemistry Research.

[30] (2012). Uniform mesoporous ZSM-5 single crystals catalyst with high resistance to coke formation for methanol deoxygenation. Microporous and Mesoporous Materials.

[31] (1992). Ordered mesoporous molecular sieves synthesized by a liquid-crystal template mechanism. Nature.

[32] (1997). Characterization of Porous Silica Templated by Surfactants. Journal of Physical Chemistry B.

[33] (1999). Composites of micro- and mesoporous materials: Simultaneous syntheses of MFI/MCM-41 like phases by a mixed template approach. Microporous and Mesoporous Materials.

[34] (2011). Hierarchical mesoporous zeolites: Direct self-assembly synthesis in a conventional surfactant solution by kinetic control over the zeolite seed formation. Chemistry -A European Journal.

[35] (2016). Synthesis of nano-SSZ-13 and its application in the reaction of methanol to olefins. Catalysis Science and Technology.

[36] (2017). Synthesis of ZSM-5 with hierarchical porosity: In-situ conversion of the mesoporous silica-alumina species to hierarchical zeolite. Microporous and Mesoporous Materials.

[37] (2010). One-step synthesis of hierarchical pentasil zeolite microspheres using diamine with linear carbon chain as single template. New Journal of Chemistry.

[38] (2017). Steam catalytic cracking of heavy naphtha (C12) to high octane naphtha over B-MFI zeolite. Applied Catalysis B: Environmental.

[39] (2017). TPABr-grafted MWCNT as bifunctional template to synthesize hierarchical ZSM-5 zeolite. Materials Letters.

[40] (1944). Surfaces of solids. XIII. A vapor adsorption method for the determination of the area of a solid without the assumption of a molecular area, and the areas occupied by nitrogen and other molecules on the surface of a solid. Journal of the American Chemical Society.

[41] (1951). The determination of pore volume and area distributions in porous substances. I. Computations from nitrogen isotherms. Journal of the American Chemical Society.

[42] (1986). Catalytic and acidic properties of boron pentasil zeolites. Studies in Surface Science and Catalysis.

[43] (2017). An NMR Crystallographic Investigation of the Relationships between the Crystal Structure and 29Si Isotropic Chemical Shift in Silica Zeolites. Journal of Physical Chemistry C.

[44] (2018). Identifying the effective phosphorous species over modified P-ZSM-5 zeolite: A theoretical study. Physical Chemistry Chemical Physics.

[45] (1994). Mechanism of structure direction in the synthesis of Si-ZSM-5: An investigation by intermolecular 1H-29Si CP MAS NMR. The Journal of Physical Chemistry.

[46] (2007). Effect of boron substitution in chabazite framework: IR studies on the acidity properties and reactivity towards methanol. Journal of Physical Chemistry C.

[47] (1985). 5 containing boron instead of aluminium atoms in the framework. Zeolites.

[48] (2015). Physisorption of gases, with special reference to the evaluation of surface area and pore size distribution (. IUPAC Technical Report). Pure and Applied Chemistry.

